# Impact of selective reporting of antimicrobial susceptibility testing report on clinicians’ prescribing behavior of antibiotics

**DOI:** 10.3389/fphar.2023.1225531

**Published:** 2023-09-28

**Authors:** Ying Wang, Xinping Zhang, Qian Zhou, Xiaojun Xu

**Affiliations:** ^1^ Nanjing Drum Tower Hospital Affiliated to Nanjing University Medical School, Nanjing, Jiangsu, China; ^2^ School of Medicine and Health Management, Tongji Medical College, Huazhong University of Science and Technology, Wuhan, Hubei, China; ^3^ Wuhan Children’s Hospital affiliated to Tongji Medical College of Huazhong University of Science and Technology, Wuhan, Hubei, China; ^4^ The First Affiliated Hospital of Gannan Medical University, Ganzhou, Jiangxi, China

**Keywords:** antimicrobial susceptibility testing (AST) results, selective reporting, prescribing behavior, appropriateness rate of prescription, antibiotic prescription

## Abstract

**Background:** Selective reporting has important value in antibiotic management. The purpose of this study was to explore the impact of AST selective reporting on prescribing behavior, so as to provide evidence for the implementation and improvement of selective reporting policies in microbiology laboratories at home and abroad.

**Methods:** A cross-sectional study was conducted in a teaching tertiary hospital in China in July 2021. We designed selective reports and routine reports for urinary tract infections caused by *Escherichia coli* and lower respiratory tract infections caused by *Pseudomonas aeruginosa*. Questionnaires were conducted among participants by case vignettes, and 116 valid questionnaires were collected. The appropriateness rate of antibiotic prescription and the prescription rate of drug-resistant antibiotics, cephalosporins, fluoroquinolones, and carbapenems were calculated and compared between the selective reporting group and the routine reporting group in each case.

**Results:** In most cases, we found that AST selective reporting could increase the appropriateness rate of antibiotic prescription (*p* < 0.05) and reduce the drug-resistant antibiotic prescription rate (*p* < 0.01), cephalosporin drug prescription rate (*p* < 0.05) and fluoroquinolone drug prescription rate (*p* < 0.01). Although the difference in carbapenems prescription rate was not significant, selective reporting could reduce the number of its prescriptions to some extent.

**Conclusion:** AST selective reporting can help promote the appropriate use of antibiotics and reduce the use of broad-spectrum antibiotics. It is suggested to develop scientific and effective selective reporting practices and strengthen the two-way communication between clinicians and microbiology laboratories, thereby enabling microbiology laboratories to play a more important role in clinical antimicrobial management.

## 1 Introduction

Improving the rationality of antimicrobial prescriptions by clinicians is a key measure to control the current serious problem of antimicrobial resistance (Jee et al., 2018). AST result is an important basis for clinical decision-making because it can guide clinicians to prescribe antibiotics (Drobniewski et al., 2015), so the method of microbiological laboratory reporting test results, as well as selective reports and instructions on how to interpret the results, could affect clinicians’ prescriptions (Morency-Potvin et al., 2016).

However, there are many problems in the current AST reports, such as the current inconsistency between the drugs listed in the AST report and the list of drugs in the pharmacy of hospitals, too many or too few drugs reported susceptibility, and the high error rate of AST report information due to the complex susceptibility test methods, which will lead to overload in the interpretation of drug susceptibility test reports and ultimately leading to incorrect diagnosis and irrational antibiotic prescription (Graham et al., 2019; Wang et al., 2021).

In order to directly and effectively solve the problem caused by laboratory reporting overload, the European Centre for Disease Prevention and Control (ECDC), the Infectious Diseases Society of America (IDSA), and the American Society for Clinical and Laboratory Standards (CLSI) all recommend to use selective reporting of AST, that is, antibiotics are tested in the same way, but focusing on encouraging the reporting of those antimicrobial drugs and narrow-spectrum antimicrobials that are suitable for the condition, or not reporting in some scenarios, which means the laboratory only selectively reports the most direct and effective results so as to better guide and standardize antimicrobial prescribing behavior (Castro-Sánchez et al., 2018; Weinstein and Lewis, 2020; Barlam et al., 2016). Selective reporting has been incorporated into the CLSI as an important part of antimicrobial stewardship and is used as the standard of medicine by national health authorities in Ireland (SARI, 2014), the United States (Pulcini et al., 2017), and Australia (AMSAH, 2018).

Despite this, selective reporting was not yet widely implemented in practice, a cross-sectional survey of European countries found that selective AST report was only implemented in 11 (31%) of the 36 participating countries (Pulcini et al., 2017). Probably because there is a lack of detailed guidelines on how to practice selective reporting, professionals’ capability and relevant resources are needed as well.

Scholars have conducted studies on the impact of selective AST reporting on the antimicrobial prescribing behavior of clinicians, mainly adjusting the type and quantity of antibiotics selectively reported according to specific specimens like urine (Bourdellon et al., 2017; Daley et al., 2018) and pathogens like *Staphylococcus aureus* (LestinBernstein et al., 2021) and Enterobacteriaceae, *Pseudomonas aeruginosa* ((Pulcini et al., 2017)) or drug resistance conditions, and there is heterogeneity in the current research results.

Some studies support selective AST reporting can promote rational antimicrobial prescribing, (Daley et al., 2018), found that the rational prescribing rate of antibiotics in the non-reporting selective reporting group was significantly higher than that in the general reporting group. (Leis et al., 2014; Howell-Jones et al., 2008). also confirmed this conclusion in asymptomatic bacteriuria and venous leg ulcer studies. (Langford et al., 2016). found AST results that did not report the sensitivity of Enterobacteriaceae to ciprofloxacin could reduce the prescription rate of ciprofloxacin by 55%. (Al-Tawfiq et al., 2015). reported AST results selectively based on guidelines and local drug resistance and found an increase in the rate of rational prescription of antibiotics and a decrease in the infection rate of *C. difficile*. (McNulty et al., 2011). switched amoxicillin/clavulanate to cephalexin in the AST report for selective reporting and found that amoxicillin/clavulanate prescription rates were reduced by nearly 70%.

While some studies found that selective AST reporting does not have a significant effect on antimicrobial prescriptions. (Papanicolas et al., 2017). found that unreported selective reporting, while effective in reducing antimicrobial prescribing behavior intention in the outpatient group, was not significant in the inpatient group. In a U.S. study, failure to report selective reporting of broad-spectrum antimicrobials in AST when narrow-spectrum antimicrobials are sensitive does not reduce the rate of prescription of broad-spectrum antimicrobials (Barnes, 1980; Smoke et al., 2019) developed principles for reporting antimicrobial resistance for each isolated strain, and selective reporting of antimicrobials for different specific antimicrobial resistance and special populations within the scope of selected antimicrobials, but there was no significant difference in intravenous prescribing rates for broad-spectrum antimicrobials before and after the intervention in seven hospitals.

The heterogeneity of research results may be attributed to significant variations in existing research models and evaluation methods. On the one hand, the inconsistency in the method of selective reporting leads to differences in reducing and substituting types and quantities of antibiotics. On the other hand, the outcome indicators selected for the studies were different and were mainly limited to the rationality of antimicrobial use or the usage rate of specific antibiotics.

Therefore, the effect of selective reporting on antimicrobial prescribing behavior needs to be further explored, particularly with a focus on specific types of infections and comprehensive analysis of the results of various antimicrobial drug usage indicators. Our study will concentrate on specific pathogen infection types and integrate various outcome measures of antibiotic usage to analyze the impact of selective reporting on clinicians’ prescribing behavior, in order to provide evidence for the formulation and implementation of selective AST reporting at home and abroad.

## 2 Materials and methods

### 2.1 Study design and participants

A cross-sectional study was conducted in a teaching tertiary hospital in Ganzhou, Jiangxi, China in July 2021.

The participant inclusion criteria were as follows: 1) Clinicians who have the right to prescribe antibiotics or senior interns with extensive prescribing-assistant experience in prescription; 2) All clinicians on duty during the on-site investigation; 3) Clinicians who can understand and fill out questionnaires.

Two types of infection were included in this study: urinary tract infection caused by *Escherichia coli* and lower respiratory tract infection caused by *P. aeruginosa*. These were the most common infection scenarios with a high frequency of prescription of antibiotics in hospitals based on relevant studies on the clinical use of antibiotics and consultation with clinicians.

Questionnaires were developed according to the two types of infection respectively and were distributed to participants according to their departments.

Strains in the research hospital were identified using the VITEK2-compact microbial identification system (BioMérieux, France). *In vitro* susceptibility testing was carried out by Kirby-Bauer disk diffusion, and the interpretation standards and quality control requirements followed the CLSI guidelines (CLSI, 2021).

### 2.2 Survey instrument

Questionnaires were developed by a multidisciplinary team of experts in infectious diseases, microbiology, public health, and clinicians, consisting of two parts:

Part One: Personal information and basic information, including gender, age, professional title, department, and views on the selective report.

Part Two: Case vignettes. This part is divided into two volumes according to urinary tract infection and lower respiratory tract infection. Case vignettes were designed based on the type of infection.

Five cases were designed in urinary tract infection: 1) Asymptomatic bacteriuria 2) Acute simple cystitis (completely susceptible) (3) Acute simple cystitis (ESBL positive) 4) Acute simple pyelonephritis (completely susceptible) 5) Complicated urinary tract infection.

Four cases were designed in lower respiratory tract infection: 1) *P. aeruginosa* colonization 2) Acute exacerbation of the chronic obstructive pulmonary disease 3) Bronchiectasis combined with infection 4) Hospital-acquired pneumonia.

### 2.3 Selective reporting

Two ways of selective reporting were considered in this study: 1) Do not report AST results when antibiotic therapy was unnecessary such as bacterial colonization. 2) Reduce the type or number of antibiotics reported, such as reducing the number of broad-spectrum antimicrobials reported if the bacteria are sensitive, and encourage reporting of narrow-spectrum antibiotics.

Clinicians can always request the microbiology laboratory to provide a complete AST report when they have questions about the selective report.

The list of antibiotics in the routine report and the principle of selective reporting of antibiotics were all referred to the Performance Standard of Antimicrobial Susceptibility Test 2021 (CLSI M100) released by the Clinical and Laboratory Standards Institute (CLSI, 2021).

Examples of two selective reporting applied to the case vignettes are as follows.

#### 2.3.1 Do not report antimicrobial susceptibility testing results

(1) In urinary tract infection, for asymptomatic female patients with bacteriuria, the possibility of colonization was considered and antibiotics were not indicated, so the routine report was replaced with selective reporting, which noted “This positive urine culture may represent asymptomatic bacteriuria or urinary tract infection. If a urinary tract infection is clinically suspected, call the microbiology laboratory for identification and routine AST results.”.
(2) In lower respiratory tract infection, the colonization of *P. aeruginosa* in patients with a history of lung disease is also considered not to require antibiotic treatment, so the routine report was replaced with selective reporting, which noted “This positive sputum culture may represent the colonization of *P. aeruginosa*. If a lung infection is clinically suspected, call the microbiology laboratory for identification and routine AST results.”.

#### 2.3.2 Reduce the type or number of antibiotics reported

This way of selective reporting mainly refers to the principle of selective reporting of AST reports of various bacteria in the ABCU group recommended in the CLSI M100 guideline to reduce the number of reported antibiotics. In addition, we only reported AST results for no more than 6 antibiotics, including those that have been used empirically.

**FIGURE 1:**
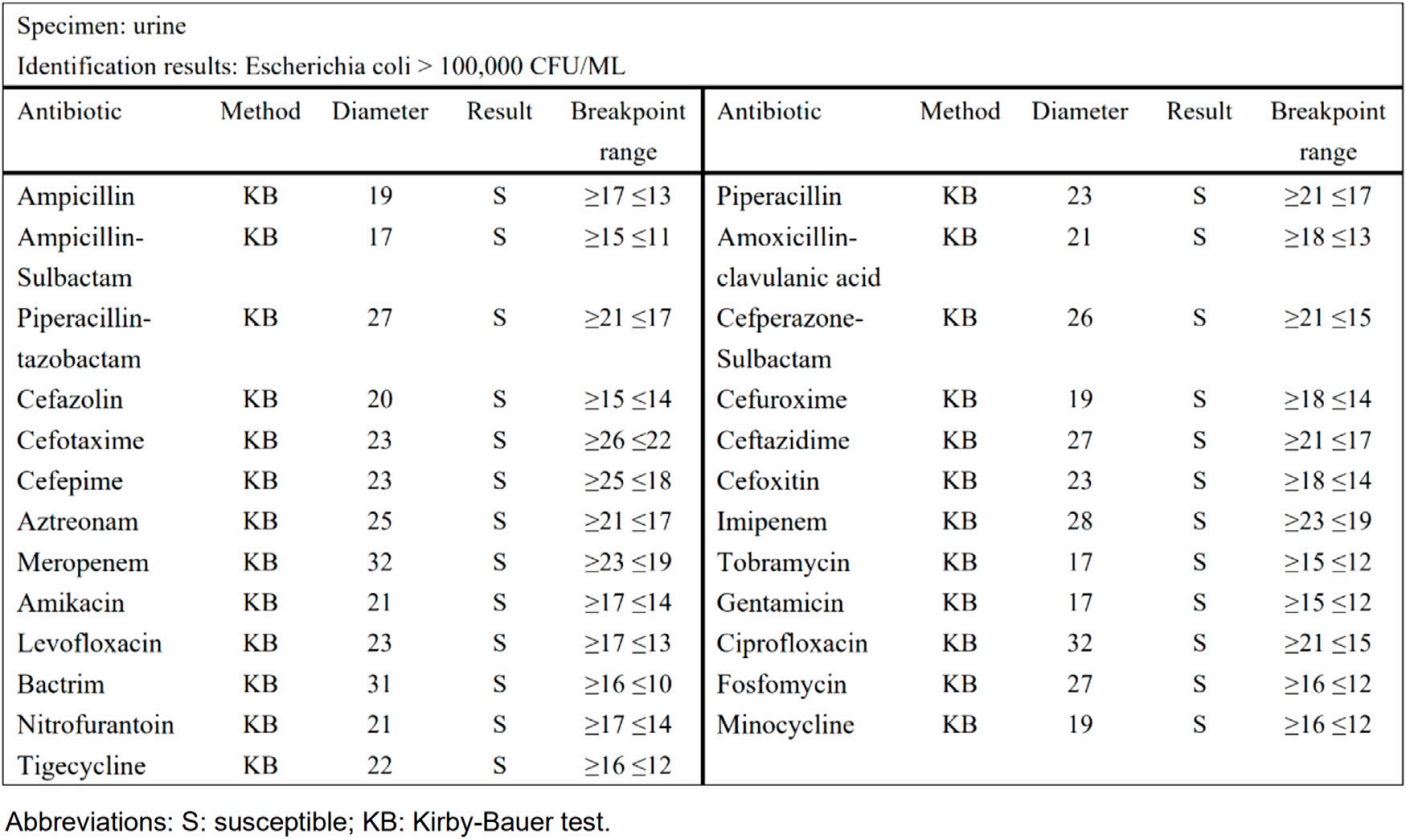
The routine report of urinary tract infection-acute simple cystitis.

**FIGURE 2:**
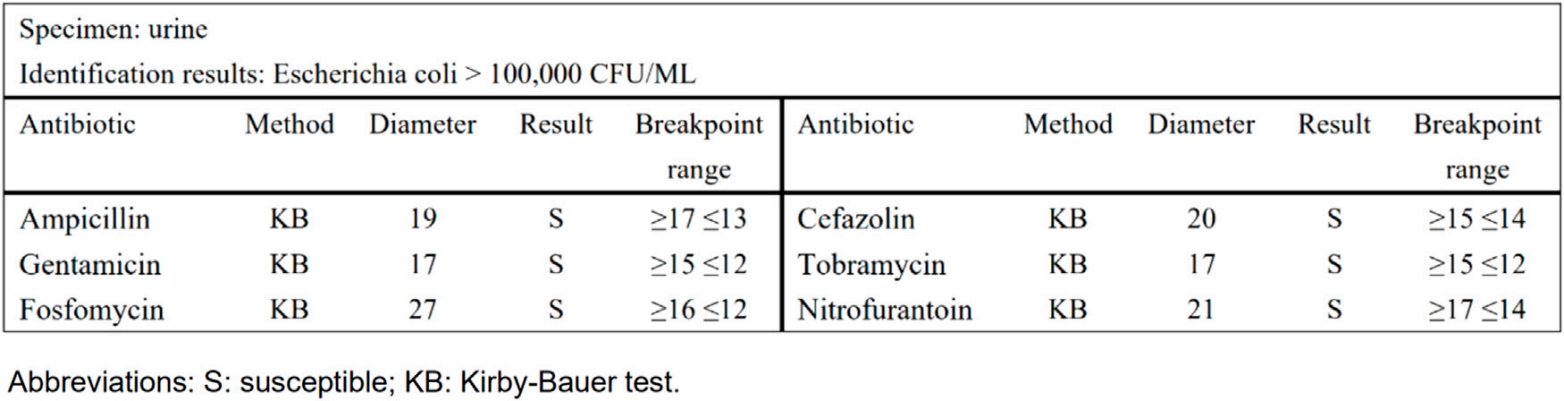
The selective report of urinary tract infection-acute simple cystitis.

Taking urinary tract infection-acute simple cystitis as an example, the routine report (Figure 1) reported 25 drugs in a routine test, while selective reporting reported 6 drugs in accordance with the ABCU principle (Figure 2), as follows.

### 2.4 Outcomes

Our primary outcome was the appropriateness rate of antibiotic prescriptions, based on the available AST results. An antibiotic prescription was considered to be appropriate if it adhered to the criterion of prescription appropriateness. The secondary outcome was the prescription rate of drug-resistant antibiotics, cephalosporins, fluoroquinolones, and carbapenems.

### 2.5 The criterion of prescription appropriateness

The criterion of prescription appropriateness was formulated by referring to the guideline (Guidelines for Clinical Application of Antibiotics (2015 edition)) (National Health and Family Planning Commission of the People’s Republic of China, 2015) and through multidisciplinary group discussion as follows.

(1) In the case of not reporting AST results, the appropriate prescription of antibiotics should be non-prescription antibiotics.
(2) In the case of reducing the type or number of antibiotics reported, the appropriate prescription should meet three conditions: 1) prescription of antibiotic is necessary; 2) The prescription of antibiotics meets the requirements of the guidelines (Detailed criterion for appropriate prescription of each case can be seen in the supplement); 3) The antibiotics prescribed are drug-susceptible.

### 2.6 Statistics analysis

In this study, IBM SPSS Statistics version 26 was used to complete all the data analysis and processing. Firstly, all the data were collected through a self-reported questionnaire. Secondly, descriptive analysis was used to describe the general characteristics of the study population and the prescription rate of antibiotics. Thirdly, the prescription rate of antibiotics between the routine and selective report of each case was compared using a chi-square test or Fisher’s exact test as appropriate. All tests were two-tailed, and a *p*-value of 0.05 was considered statistically significant.

## 3 Results

### 3.1 Characteristics of clinicians and their views on selective reporting of antimicrobial susceptibility testing results

A total of 116 questionnaires were collected. The participants’ ages ranged from 20 to 58 years old (33.35 ± 7.72), with 5 missed age information; a majority of the sample was males (70%, 60.34%), and 2 missed professional title information. With regard to clinicians’ views on selective reporting, 62.93% of doctors believe that selective reporting in the scenario simulation is more helpful in choosing antibiotics, and 79.31% of doctors believe that selective reporting should be applied to clinical practice.

### 3.2 Outcomes

All the outcomes about the prescription rates and numbers of antibiotics for each case of urinary tract infection and lower respiratory tract infection can be seen in Table 1, Table 2, Table 3, and Table 4.

**TABLE 1:** The prescription rates of antibiotics of cases of urinary tract infection (N = 53) (n,%).

Case	Group	Appropriateness rate	Drug-resistant antibiotics	*Cephalosporins*	*Cephalosporins* (exclude *cefazolin*)	*Fluoroquinolones*	*Carbapenems*
Case 1	Routine	14 (26.42)	-	10 (25.64)	9 (23.08)	10 (25.64)	0 (0.00)
	Selective	37 (69.81)	-	4 (25.00)	4 (25.00)	4 (25.00)	2 (12.50)
	P	***	-	1	1	1	0.081
Case 2	Routine	0 (0.00)	-	11 (24.44)	8 (17.78)	20 (44.44)	1 (2.22)
	Selective	4 (7.55)	-	18 (45.00)	1 (2.50)	0 (0.00)	0 (0.00)
	P	0.126	-	*	*	***	1
Case 3	Routine	8 (15.09)	13 (30.23)	9 (20.93)	8 (18.60)	8 (18.60)	2 (4.65)
	Selective	25 (47.17)	2 (4.26)	0 (0.00)	0 (0.00)	0 (0.00)	0 (0.00)
	P	***	***	**	**	**	0.225
Case 4	Routine	36 (67.92)	-	2 (4.44)	1 (2.22)	30 (66.67)	2 (4.44)
	Selective	30 (56.60)	-	6 (12.77)	0 (0.00)	29 (61.70)	0 (0.00)
	P	0.229	-	0.296	0.489	0.62	0.237
Case 5	Routine	36 (67.92)	6 (12.00)	5 (10.00)	5 (10.00)	1 (2.00)	4 (8.00)
	Selective	35 (66.04)	4 (8.33)	0 (0.00)	0 (0.00)	4 (8.33)	0 (0.00)
	P	0.836	0.79	0.073	0.056	0.334	0.136

***: *p*≤0.001; **: *p* ≤ 0.01; *: *p* ≤ 0.05.

**TABLE 2:** The prescription rates of antibiotics of cases of lower respiratory tract infection (N = 63) (n,%).

Case	Group	Appropriateness rate	Drug-resistant antibiotics	*Cephalosporins*	*Fluoroquinolones*	*Carbapenems*
Case 1	Routine	12 (19.05)	-	13 (25.49)	13 (25.49)	0 (0.00)
	Selective	50 (79.37)	-	2 (15.38)	1 (7.69)	0 (0.00)
	P	***	-	0.688	0.313	-
Case 2	Routine	47 (74.60)	-	6 (11.76)	33 (64.71)	3 (5.88)
	Selective	50 (79.37)	-	13 (25.00)	25 (48.08)	0 (0.00)
	P	0.525	-	0.083	0.089	0.234
Case 3	Routine	49 (77.78)	10 (16.67)	19 (31.67)	4 (6.67)	32 (53.33)
	Selective	58 (92.06)	0 (0.00)	19 (32.20)	3 (5.08)	38 (64.41)
	P	*	**	0.95	1	0.22
Case 4	Routine	46 (73.02)	9 (15.25)	1 (1.69)	42 (71.19)	6 (10.17)
	Selective	51 (80.95)	8 (13.11)	0 (0.00)	40 (65.57)	2 (3.28)
	P	0.29	0.737	0.492	0.509	0.251

***: *p*≤0.001; **: *p* ≤ 0.01; *: *p* ≤ 0.05.

**TABLE 3:** The prescription numbers of antibiotics of cases of urinary tract infection (N = 53).

Antibiotic	Case 1		Case 2		Case 3		Case 4		Case 5	
	Routine	Selective	Routine	Selective	Routine	Selective	Routine	Selective	Routine	Selective
Ampicillin	4		6	(16)	3	1	3	(7)	2	
Piperacillin	1				1	1			2	
Ampicillin-Sulbactam	5	2	2		4	(9)	4	1	3	(11)
Amoxicillin-clavulanic acid	2	2	1		4	(12)			5	(22)
Piperacillin-tazobactam	5		3		9	(10)	3		19	1
Cefperazone-Sulbactam	2		1		3	8	1		4	
Cefazolin	1		3	(17)	1		1	(6)		
Cefuroxime	3		1							
Cefotaxime	5	4	6	1	2		1			
Ceftazidime	1		1		5				5	
Cefepime					1					
Cefoxitin										
Aztreonam										
Imipenem							1			
Meropenem		2	1		2		1		4	
Tobramycin						(2)				
Amikacin									1	
Gentamicin				(2)				(3)		(3)
Levofloxacin	9	4	19		6		30	(29)	1	(4)
Ciprofloxacin	1		1		2					
Bactrim	1	2							1	(1)
Fosfomycin				(1)		(4)		(1)	1	(5)
Nitrofurantoin				(3)					1	
Minocycline										

“()” numbers indicate antibiotics that were reported in the selective report.

**TABLE 4:** The prescription numbers of antibiotics of cases of lower respiratory tract infection (N = 63).

Antibiotic	Case 1		Case 2		Case 3		Case 4	
	Routine	Selective	Routine	Selective	Routine	Selective	Routine	Selective
Piperacillin	11	2	2		2		1	
Piperacillin-tazobactam	3	2	4	(14)	1			2
Cefperazone-Sulbactam	11	5	3		5		3	2
Cefperazone	1		2		1			
Ceftazidime	12	2	4	(13)	17	(19)	1	
Cefepime					1			
Aztreonam	1		2					
Imipenem			1		12	(18)	5	1
Meropenem			2		20	(20)	1	1
Amikacin		2					2	1
Gentamicin				(2)			5	(12)
Tobramycin							5	(6)
Levofloxacin	6		4		1	(1)	2	(1)
Ciprofloxacin	7	1	29	(25)	3	(2)	40	(39)
Bactrim								
Minocycline								

“()” numbers indicate antibiotics that were reported in the selective report.

#### 3.2.1 The appropriateness rate of antibiotic prescriptions

In the case of do not report AST results, the appropriate prescription rate of the selective reporting group was significantly higher than that of the routine reporting group in case 1 of urinary tract infections (69.81% vs. 26.42%, *p* < 0.001) and case 1 of respiratory tract infections (79.37% vs. 19.05%, *p* < 0.001).

In the case of reducing the type or number of antibiotics reported, the appropriate prescription rate of the selective reporting group was significantly higher than that of the routine reporting group in case 3 of urinary tract infections (47.17% vs. 15.09%, *p* < 0.001) and case 3 of respiratory tract infections (92.06% vs. 77.78%, *p* < 0.05). The appropriate prescription rate of the selective reporting group was also higher than that of the routine reporting group in case 2 of urinary tract infections (7.55% vs. 0.00%, *p* = 0.126) and case 2 of respiratory tract infections (79.37% vs. 74.60%, *p* = 0.525) and case 4 (80.95% vs. 73.02%, *p* = 0.290), but the difference was not significant. In case 4 (56.60% vs. 67.92%, *p* = 0.229) and case 5 (66.04% vs. 67.92%, *p* = 0.836) of urinary tract infections, the appropriate prescription rate of the selective reporting group was lower than that of the routine reporting group, but the difference was not significant.

#### 3.2.2 The prescription rate of drug-resistant antibiotics

The selective reporting group had a significantly lower rate of drug-resistant antibiotic prescription than the routine reporting group in case 3 of urinary tract infections (4.26% vs. 30.23%, *p* < 0.001) and case 3 of respiratory tract infections (0% vs. 16.67%, *p* < 0.01). In case 5 of urinary tract infections (66.04% vs. 67.92%, *p* = 0.836) and case 4 of respiratory tract infections (13.11% vs. 15.25%, *p* = 0.737), the rate of drug-resistant antibiotic prescription in the selective reporting group was also lower than that in the routine reporting group, but the difference was not significant.

#### 3.2.3 The prescription rate of cephalosporins

In cases of urinary tract infection, the selective reporting group had a significantly lower prescription rate of cephalosporins compared to the routine reporting group in case 3 (0.00% vs. 20.93%, *p* < 0.01). However, in case 2, the selective reporting group had a significantly higher prescription rate of cephalosporins than the routine reporting group (45.00% vs. 24.44%, *p* < 0.05), but the prescription rate of other cephalosporins excluding cefazolin (first-generation cephalosporin) was significantly lower in the selective reporting group compared to the routine reporting group (2.50% vs. 17.78%, *p* < 0.05). In case 4, the selective reporting group also had a higher prescription rate of cephalosporins than the routine reporting group (12.77% vs. 4.44%, *p* = 0.296), but the prescription rate in the selective reporting group was lower than that in the routine reporting group (0% vs. 2.22%, *p* = 0.489) after excluding cefazolin. In cases 1 (25% vs. 25.64%) and five (0.00% vs. 10%), the prescription rate of cephalosporins in the selective reporting group was also lower than that in the routine reporting group, the difference was not significant.

For respiratory tract infections, the prescription rate of cephalosporins in the selective reporting group was higher than that in the routine reporting group in case 2 (25% vs. 11.76%, *p* = 0.083) and case 3 (32.2% vs. 31.67%, *p* = 0.950), while it was lower in the selective reporting group compared to the routine reporting group in case 1 (15.38% vs. 25.49%, *p* = 0.668) and case 4 (0% vs. 1.69%, *p* = 0.492).

#### 3.2.4 The prescription rate of fluoroquinolones

In cases of urinary tract infection, the selective reporting group had significantly lower prescription rates of fluoroquinolones compared to the routine reporting group in case 2 (0% vs. 44.44%, *p* < 0.001) and case 3 (0% vs. 18.60%, *p* < 0.01). The selective reporting group also had lower prescription rates of fluoroquinolones compared to the conventional reporting group in case 1 (25% vs. 25.64%) and case 4 (61.70% vs. 66.67%), but the differences were not significant. In addition, the selective reporting group had a higher prescription rate of fluoroquinolones compared to the routine reporting group in case 5 (8.33% vs. 2%, *p* = 0.334).

In cases of lower respiratory tract infection, although the differences were not significant, the selective reporting group had lower prescription rates of fluoroquinolones compared to the routine reporting group in all cases.

#### 3.2.5 The prescription rate of carbapenems

In cases of urinary tract infection, there were only slight changes in the prescription of carbapenems, and the differences were not significant. However, in the selective reporting group, there was a decrease compared to the routine reporting group in cases 2 (0% vs. 2.22%), case 3 (0% vs. 4.65%), case 4 (0% vs. 4.44%), and case 5 (0% vs. 8%), while case 1 (12.5% vs. 0%) showed an increase.

In cases of respiratory tract infection, the prescription numbers were higher than in urinary tract infections, but the differences were still not significant. Among them, case 2 (0% vs. 5.88%) and case 4 (3.28% vs. 10.17%) in the selective reporting group showed a decrease compared to the routine reporting group. Case 3 (64.41% vs. 53.33%) in the selective reporting group showed an increase compared to the routine reporting group, but the difference was not significant.

## 4 Discussion

This study found that selective reporting can promote the appropriate prescription of antibiotics, not only improving the appropriate prescription rate of antibiotics but also reducing the prescription rate of drug-resistant antibiotics.

Firstly, selective reporting can significantly improve the appropriateness rate of antimicrobial prescriptions by providing clinicians with a concise and effective drug list based on the principle of selective reporting and clinical application guidelines for antibiotics, similar results were obtained in the studies by (Coupat et al., 2013; Bourdellon et al., 2017). Although the difference in the appropriateness rate of antimicrobial prescriptions between the selective reporting group and the routine reporting group was not significant in case 2 of urinary tract infection and cases 2 and 4 of respiratory tract infection, an increase in the appropriateness rate was still observed. The slight decrease in the rationality rate in cases 4 and 5 of urinary tract infection may be due to the fact that although ampicillin and cephalothin were included in the selective reporting list recommended by CLSI-M100, they were not on the list of recommended drugs for clinical use of antibiotics in China, which leads to a certain degree of decrease in the appropriateness rate. This indicates that there is a certain difference between the antibiotics recommended for selective reporting in the reference performance standard guidelines for antimicrobial susceptibility testing (CLSI-M100) in China and the antibiotics recommended for clinical use of antibiotics in China.

Additionally, we found that in the case of reducing the type or number of antibiotics reported, selective reporting improved the appropriateness rate of prescriptions for respiratory tract infections and partially improved the appropriateness rate for urinary tract infections. This suggests that the overall application effect of selective reporting on the appropriate prescription of antibiotics for respiratory tract infections is better than urinary tract infections, which may be due to a higher degree of conformity between the antibiotics recommended for selective reporting in CLSI-M100 and those recommended for clinical use of antibiotics in China for respiratory tract infections compared to urinary tract infections. Therefore, the application of selective reporting in China needs to take into account factors such as the use of antibiotics in China and the habits of different medical institutions in different regions and consider the specific situations of different infections to better play its role in antimicrobial management.

Selective reporting can also significantly reduce the prescription rate of drug-resistant antibiotics. The selection of drug-resistant antibiotics will not only be ineffective in clinical treatment but also may aggravate bacterial drug resistance. We found that the reduction of the prescription rate of drug-resistant antibiotics in the selective reporting of urinary tract infections was greater than that of respiratory tract infections, because the selective reporting is to give priority to the reporting of sensitive first-line drugs, drug-resistance antibiotics were reported only if the same drugs were resistant and there is no alternative sensitive drug. The prescription rate of drug-resistance antibiotics for urinary tract infections was higher than that for respiratory infections in this study, so selective reporting of drug-resistance reduction was also better.

However, due to the differences between the antibiotics recommended for selective reporting in CLSI-M100 and those recommended for clinical use of antibiotics, selective reporting can only reduce the prescription of drug-resistant antibiotics to a certain extent. Furthermore, when the drug-resistance situation is more complex in cases such as multi-drug resistance, the effect of selective reporting on reducing the prescription rate of drug-resistant antibiotics will become smaller.

This study also found that selective reporting could reduce the prescription of three broad-spectrum antibiotics: cephalosporins (other than cefazolin), fluoroquinolones, and carbapenems. The problem of antimicrobial resistance is becoming more and more serious under the widespread use of these three types of antibiotics whose prescription should be controlled to reduce antimicrobial resistance as much as possible.

The results showed that selective reporting could significantly reduce the prescription rate of cephalosporins (except cefazolin), and the rate in case 3 of urinary tract infection was significantly lower than that reported by routine reporting, and also decreased to a certain extent in case 1 and case 5. This is similar to the results of (Bourdellon et al., 2017). Similarly, in respiratory tract infection, the prescription rate of ceftazidime increased in case 2 and case 3, possibly because ceftazidime was listed in selective reporting as a sensitive drug, and ceftazidime was also recommended in clinical application guidelines for the two cases. but the rate decreased in case 4 because AST results showed multi-drug resistance and there was no cephalosporin in the selective reporting, so the prescription rate was reduced to 0%. Overall, in the case of no multi-drug resistance, the prescription rate of urinary tract infection and respiratory tract infection in the selective reporting group would be higher than that in the routine reporting group because cefazoline and ceftazidime appeared in the selective reporting group. While in the case of multi-drug resistance, cephalosporin was not listed in the selective reporting group because of drug resistance. Therefore, the prescription rate of cephalosporin in the selective reporting group was 0%.

Selective reporting can also significantly reduce the prescription rate of fluoroquinolones. (Coupat et al., 2013; Langford et al., 2016). also found a similar result. For urinary tract infections, fluoroquinolone prescriptions decreased to 0 in both case 2 and case 3 in selective reporting groups, mainly because both cases were simple urinary tract infections with more bacterial-sensitive drugs, and fluoroquinolones were not included in the selective reporting. Therefore, clinicians may prescribe fluoroquinolones in routine reporting groups due to experience. But selective reporting makes clinicians choose more narrow-spectrum antibiotics; In addition, we found that selective reporting of fluoroquinolone prescribing rates decreased in all cases of respiratory tract infections, but to a lesser extent than urinary tract infections. On the one hand, fluoroquinolones are commonly used in the clinical treatment of respiratory infections, especially when fluoroquinolones are used empirically, even if they are not included in selective reporting. Clinicians may also choose to prescribe fluoroquinolones out of habit, so selective reporting could only reduce the prescription of fluoroquinolones in respiratory infections to a certain extent.

Selective reporting also reduced the prescription rate of carbapenems. Although the change was not significant, reductions were observed in almost all cases. The low number of prescriptions of carbapenems in these cases may be related to the prescribing habits of clinicians, and selective reporting has the effect of reducing carbapenems in these cases. We also observed an increase in the prescription of carbapenems in the case of not reporting AST results, which suggests that clinicians may prefer to prescribe carbapenems for treatment when they do not have clear AST results but suspect a urinary tract infection. In addition, the prescription of carbapenems was much higher in case 3 of respiratory tract infection than in other cases, and the prescription rate of the selective reporting group was higher than that of the routine reporting group, which may be related to the situation of the cases themselves. For multi-drug resistant cases of bronchiectasis combined with infection, carbapenems were all sensitive drugs and were reported in the selective reporting. Clinicians were more likely to prescribe carbapenems when the report listed fewer drugs, in which case selective reporting could not reduce carbapenem prescriptions. Consequently, in the case of multi-drug resistance, if there are sensitive carbapenems in the selective report, the selective report will not reduce the prescription of carbapenems.

According to the above analysis, our study supports that selective reporting can improve the appropriate prescription of antibiotics, but only up to a point, since our selective reporting is based on the method recommended by CLSI M100, and our criterion of prescription appropriateness is to refer to the local antibiotic application guidelines, the appropriate prescription rate only changed significantly in some cases. The more complex the cases such as multi-drug resistance, the smaller the change in the appropriateness rate of prescription. Whereas many countries have not yet undertaken or are in the initial stages of undertaking selective reporting (Pulcini et al., 2017), we think similar discrepancies exist in other parts of the world as well. Because there is still a gap between the selective reporting method recommended by CLSI and the local clinical application of antibiotics in different countries or regions. Especially for countries or regions where selective reporting is being introduced for the first time, the existence of such differences may be beyond the scope of clinicians and affect clinical outcomes.

In addition, the accuracy of the measurement method of AST results would also have an impact on the effect of selective reporting, because it may yield inaccurate results for pathogens exhibiting hetero resistance (Brukner and Oughton, 2020), which could affect the prescription of antibiotics and clinical outcomes. Therefore, the improvement and standardization of the AST method is the guarantee and foundation to promote the implementation of selective reporting.

To better promote the application of selective reporting and give full play to its role in the management of antibiotics, we suggest that each medical institution from countries or regions where selective reporting is being introduced for the first time should form an antimicrobial management team composed of microbiology laboratories, clinical departments, pharmacy departments, and other disciplines. Then, the selective reporting operation norms and guidelines applicable to each bacteria or various infections should be formulated to form a multidisciplinary consensus according to the relevant guidelines for the application of antibiotics combined with the actual situation of each hospital. Thus, appropriate use of antibiotics can be promoted and antimicrobial resistance can be slowed down.

Furthermore, the implementation of AST selective reporting does not mean that the microbiology laboratory only needs to report the selective AST results. It is not the final link of the microbiology laboratory but should be an integral part of the antibiotic prescription process. On the one hand, the selective report only provides the most direct and effective drug susceptibility results for clinicians. When clinicians have doubts about the results of the selective report or want to obtain more information, they need to communicate with the microbiology laboratory in time to obtain the laboratory’s supplementary interpretation of the report and suggestions on infection control. On the other hand, if the microbiology laboratory can understand reliable and accurate clinical information during selective reporting, it will help it to report AST results faster and more accurately. Therefore, good communication between clinicians and microbiology laboratories is recommended to promote the good implementation of selective reporting and appropriate prescription of antibiotics.

As far as we know, this is the first study conducted to evaluate the impact of selective reporting on antibiotic prescribing behavior in developing countries. The research content is consistent with present international studies, on this basis, we integrated various indicators of antibiotic prescribing outcomes from two infection scenarios to analyze the influence more comprehensively. There is a strong indication that selective reporting would be a useful tool for antimicrobial management, we encourage more countries and regions to implement selective reporting, and this study can provide some reference for them, as well as provide evidence for the improvement of selective reporting policies at home and abroad.

Our study has some limitations. Firstly, all data were collected by clinicians’ self-report, longitudinal or experimental design should be further adopted in the future to explore the causal relationship between selective AST reporting and antibiotic prescribing behavior. Secondly, the cross-sectional survey data of this study came from only one general hospital, so the generalization of the research results is limited, data can be collected from multiple medical institutions to make the results more representative in the future. Finally, as the AST results were obtained directly from the microbial laboratory, we could not verify the accuracy of the MIC value, it will be considered in our future studies.

## Data Availability

The raw data supporting the conclusion of this article will be made available by the authors, without undue reservation.
