# Supplementary material for: Impact of selective reporting of antimicrobial susceptibility testing report on clinicians’ prescribing behavior of antibiotics
Source: Front Pharmacol. 2023 Sep 28;14:1225531. doi: 10.3389/fphar.2023.1225531 (PMC10571699; doi:10.3389/fphar.2023.1225531)
Supplement: Supplementary file 1 [file Table1.DOCX]

Table 1 Urinary tract infection cases and the criterion of prescription appropriateness

| Case | Case situation | List of antibiotics that can be prescribed |
| --- | --- | --- |
| Case 1 | Asymptomatic bacteriuria | none |
| Case 2 | Acute simple cystitis  (Completely susceptible) | Nitrofurantoin、Fosfomycin Trometamol、Bactrim、  Cephalexin、Cefradine |
| Case 3 | Acute simple cystitis  (ESBL positive) | Amoxicillin-clavulanic acid、Ampicillin-Sulbactam、Nitrofurantoin、Fosfomycin Trometamol |
| Case 4 | Acute simple pyelonephritis  (Completely susceptible) | Cefamandole、Cefuroxime、Cefaclor；Cefperazone、Cefperazone-Sulbactam、Cefotaxime、Ceftazidime、Ceftizoxime、Ceftriaxone、Cefixime；Levofloxacin、Ciprofloxacin、Ampicillin-Sulbactam、Amoxicillin-clavulanic acid |
| Case 5 | Complicated urinary tract infection | Piperacillin-tazobactam、Ampicillin-Sulbactam、Amoxicillin-clavulanic acid、Ceftazidime、Cefepime、Imipenem、Meropenem、Panipenem、Biapenem、Faropenem |

Table 2 Lower respiratory tract infection cases and the criterion of prescription appropriateness

| Case | Case situation | List of antibiotics that can be prescribed |
| --- | --- | --- |
| Case 1 | Pseudomonas aeruginosa colonization | none |
| Case 2 | Acute exacerbation of the chronic obstructive pulmonary disease | Ceftazidime、Cefepime、Piperacillin-tazobactam、Cefperazone-Sulbactam、Aztreonam、Imipenem/Meropenem/ Biapenem、Ciprofloxacin、Levofloxacin、Amikacin、Tobramycin、Polymyxin E/B |
| Case 3 | Bronchiectasis combined with infection，MDR | Ciprofloxacin、Levofloxacin、Anti-pseudomonas beta-lacamides（Ceftazidime、Cefepime、Beta-lactam or beta-lactamase inhibitors、Carbapenems）±Aminoglycosides |
| Case 4 | Hospital-acquired pneumonia，MDR， | Piperacillin、Ceftazidime、Cefepime、Ciprofloxacin、Levofloxacin、Beta-lactam or beta-lactamase inhibitors or carbapenems with anti-Pseudomonas aeruginosa action ± Aminoglycosides |
